# O trem e a “senhorita espanhola”: a epidemia de gripe em Diamantina, 1918

**DOI:** 10.1590/S0104-59702023000100024

**Published:** 2023-07-10

**Authors:** Ramon Feliphe Souza

**Affiliations:** i Casa de Oswaldo Cruz Fiocruz Rio de Janeiro RJ Brasil ramon.feliphe@live.com Doutor em história das ciências e da saúde, Casa de Oswaldo Cruz/Fiocruz. Rio de Janeiro – RJ – Brasil ramon.feliphe@live.com

**Keywords:** história, gripe espanhola, epidemia, Minas Gerais, ferrovias, history, Spanish flu, epidemic, Minas Gerais, railroads

## Abstract

O artigo analisa a epidemia de gripe de 1918 em Diamantina, no interior de Minas Gerais. A partir de fontes bibliográficas e documentais, discute como o ramal ferroviário da Estrada de Ferro Vitória a Minas, inaugurado em 1914, contribuiu para a chegada da doença à cidade que, até então, era representada no discurso de suas elites como isolada e salubre. Aborda as imbricadas relações entre a expansão dos sistemas de transportes pelo interior do Brasil, o meio ambiente, o conhecimento científico e os processos saúde/doença.

## Abstract (en)

The article analyzes the influenza epidemic in 1918 in Diamantina, a town in the Brazilian state of Minas Gerais. Bibliographic and documental sources are used to investigate the influence of the Vitória-Minas railroad (Estrada de Ferro Vitória a Minas), opened in 1914, on the arrival of the disease in the town, which had until then been represented in the discourse of its elites as insalubrious and isolated. The interrelations between the spread of transportation systems across Brazil, the environment, scientific knowledge, and health-disease processes are discussed.

A epidemia de gripe de 1918 é recorrentemente destacada na literatura como uma das mais letais na história. Era outubro de 1918 quando os jornais de Diamantina, no norte de Minas Gerais, publicaram as primeiras notícias sobre a epidemia que avançava pelo mundo e já se manifestava em algumas cidades brasileiras. O clima ameno e o isolamento regional foram destacados no discurso oficial como os elementos que garantiriam o abrandamento da doença. No entanto, a epidemia avançou, desorganizou o cotidiano da cidade e fez vítimas. Nem mesmo os locais percebidos como “isolados” permaneceram ilesos à sua manifestação. O artigo analisa esse contexto a partir do município de Diamantina, onde a ferrovia da Estrada de Ferro Vitória a Minas (EFVM), inaugurada em 1914, contribuiu para a chegada da doença na cidade que era representada nos discursos de suas elites como “isolada e salubre”. O progresso e o fim do isolamento trariam também a doença.

Segundo Dall’ava e Mota (2017 , p.430), “acredita-se que a doença tenha sido trazida para o território nacional por um navio inglês, o Demerara, que passou pelos portos do Recife, Salvador e Rio de Janeiro”. Seguindo a costa litorânea ( Brito, 1997 , p.12) e partindo para o interior do país, a *influenza* teve seu avanço facilitado pelas rotas de comércio e de maior circulação de pessoas. Em Minas Gerais, um dos estados brasileiros que mais se havia beneficiado com a política ferroviária republicana, a malha ferroviária teve forte influência na proliferação da doença.

Quando a epidemia irrompeu, o país, orientado pelos ideais da República (1889), estava em efervescente busca pela imagem de moderno, e as ferrovias foram entendidas como essenciais para esse processo. Foi a partir do novo regime que ocorreu uma definição mais precisa das competências federais e estaduais em relação às concessões para a construção ferroviária ( Brasil, 1890 ). As linhas férreas, entre outras obras de infraestrutura, além de aproximar os sertões e mercados percebidos como isolados, compunham um projeto ambicioso que abrangia aspectos como: a legitimação do novo regime político, a internalização da autoridade estatal, a integração do território nacional e o melhor aproveitamento de seus recursos naturais ( Caser, 2009 ; Silva, Sá, Sá, 2015). Contudo, essas obras interagiram e produziram efeitos diversos na saúde e no espaço biofísico das regiões em que se consolidaram.

No Brasil do início do século XX, as regiões atravessadas por ferrovias foram objeto de ações sanitárias relativamente bem-sucedidas. Segundo Benchimol e Silva (2008) , os cientistas responsáveis pela manutenção das condições sanitárias nas áreas de construções ferroviárias, no período, não se limitaram a fazer apenas campanhas sanitárias, fizeram também várias observações sobre doenças como a malária, por exemplo, discriminando aspectos relacionados aos seus hospedeiros e aos ambientes em que se manifestou. Essas investigações ajudaram a conformar a medicina tropical no Brasil, que, como salientam os autores, lida com ciclos de vida de parasitas em múltiplos hospedeiros e com sinergias muito dinâmicas entre processos biológicos e os ciclos econômicos das sociedades humanas. Os autores apontam a Estrada de Ferro Madeira-Mamoré como o exemplo mais emblemático do impacto das doenças tropicais nas obras de infraestrutura associadas à modernização na época (Benchimol, Silva, 2008, p.741).

A Madeira-Mamoré, ou Ferrovia do Diabo, como ficou conhecida, pretendia ligar a Amazônia brasileira à boliviana, principalmente com base dos interesses relacionadas à exploração do látex na região. Considerando suas duas fases de implementação (1878-1879 e 1907-1912), mais de seis mil pessoas foram vitimadas pelas “moléstias reinantes” na área atravessada, principalmente pela malária ( Hardman, 1988 ). A Brazil Railway Co., empresa responsável pela linha férrea, pressionada pela opinião pública – inclusive por denúncias da imprensa internacional, publicou em 1913 um conjunto de relatórios médico-sanitários destacando a atuação médica nos campos de construção como a grande responsável pelo triunfo técnico do maquinismo e da engenharia civil daquela ferrovia. Segundo Hardman, o saber médico assegurou que a produtividade dos contingentes de operários nos campos de construção da ferrovia fosse mantida em um patamar mínimo suficiente para evitar a paralisação das obras.

Destacamos também o estudo de Sutter (2005) que analisou a relação entre mudanças ambientais e o controle de doenças durante a construção do canal do Panamá, realizada pelos EUA entre 1904 e 1914. Segundo o autor, as intervenções ocasionadas pela construção do canal contribuíram para a proliferação de vetores e, consequentemente, para a alta incidência de doenças entre os trabalhadores empregados nas obras. Os entomologistas envolvidos na construção do canal perceberam que o controle dos vetores geralmente incluía o ataque a incubatórios criados pelas mudanças ambientais e sociais provocadas pelas obras. Sutter argumenta que, embora esses profissionais concordassem com objetivos mais amplos do imperialismo estadunidense na região, eles não puderam ignorar a percepção de que o controle dos vetores tinha muito a ver com a neutralização dos impactos ambientais causados pelas próprias atividades dos EUA no Panamá. Essa percepção, segundo o autor, subverteu o discurso oficial sobre a natureza dos trópicos, muitas vezes relacionado a doenças como malária e febre amarela. Na mesma direção, citamos a análise de Carter (2007) que observou como o controle da malária na Argentina se ancorou em paisagens muitos específicas: os pântanos do noroeste daquele país. Segundo o autor, apesar das medidas de “saneamento ambiental” nessas regiões, especialmente a drenagem de áreas úmidas, os mosquitos do gênero anofelina, principal vetor da doença na região, adaptaram-se bem à engenharia sanitária implementada. Destacamos também o estudo de Huffard Jr. (2013) sobre ferrovias no sul dos EUA. O autor demonstra que, no fim do século XIX, ocorreram manifestações violentas contra as empresas ferroviárias daquela região devido à percepção das comunidades sulinas – marcadas por violenta opressão contra a população afro-americana – de que as ferrovias estavam intrinsecamente ligadas à propagação da febre amarela. O conflito com as ferrovias, segundo Huffard Jr., tornou-se uma questão de sobrevivência para as populações envolvidas. O estudo chama atenção para os perigos muitas vezes ignorados entre a expansão das relações de mercado e o meio ambiente.

Certamente, as condições materiais ocasionadas pela construção do ramal de Diamantina, como os sulcos que se acumularam no solo, o desmatamento produzido ou os abrigos precários dos trabalhadores envolvidos em sua construção e manutenção – que em 1910 contava com 1.200 trabalhadores (Relatório..., 1911, p.2) –, produziram efeitos na saúde e no ambiente local e ainda precisam de mais investigações. Nossa preocupação, porém, não se reduz ao período de sua construção (1909-1914), discutimos também como, pouco depois de os trabalhos de engenharia terem sido concluídos, o pleno funcionamento da ferrovia tornou aquela parte de Minas Gerais mais sensível à epidemia de gripe de 1918 ao aproximá-la da Estrada de Ferro Central do Brasil (EFCB), uma das malhas ferroviárias mais coesas do país no período ( Campos, 2012 ).

Há estudos que indicam como as ferrovias facilitaram a disseminação da epidemia de 1918 no Brasil. Souza (2007 , p.287) analisou o modo como a gripe se infiltrou na Bahia e os diversos efeitos que provocou naquele estado. A autora afirma que, “partindo da estação da Calçada, em Salvador, a epidemia se alastrou em direção a Juazeiro, no interior baiano”. Ela argumenta que à medida que a epidemia avançava foi revelando fragilidades da sociedade baiana na República Velha, como clientelismo e nepotismo, somadas a: ausência de políticas públicas de saúde abrangentes, contínuas e eficazes; relação entre questões econômicas e a condição sanitária da capital do estado; práticas institucionais e a legislação que as presidia; e precárias condições de vida e de saúde do povo baiano (p.354). Silveira (2008 , p.79), por sua vez, analisou a relação entre a experiência da epidemia de gripe e o imaginário sobre a jovem Belo Horizonte (fundada em 1897). A nova capital mineira representou, segundo a autora, a materialização de um projeto de cidade moderna porque seria, ao mesmo tempo, salubre e higiênica. No entanto, a movimentação cotidiana de ferrovias entre Belo Horizonte e Rio de Janeiro teria tornado inevitável a chegada da doença na nova cidade. Citamos também o trabalho de Damacena Neto (2011, p.62) que, ao analisar a incidência da gripe na cidade de Goiás, afirmou que a epidemia chegou pelo caminho percorrido pela ferrovia, atingindo primeiramente as cidades do itinerário: Catalão, Ipameri e outras. Segundo o autor, nessas regiões interioranas o reconhecimento da epidemia pelo governo estadual foi tardio, assim “o envio de verbas para medidas profiláticas” demorou, e muitas pessoas sucumbiram à doença, numa situação agravada pela inexistência do serviço sanitário estadual. Em contraste, as autoridades na capital goiana tiveram tempo, argumenta o autor, de se “prevenir do mal” e organizaram uma série de medidas sanitárias que mudou o cotidiano daquela cidade (p.18).

Mesmo no período em que irrompeu a epidemia, a relação entre a expansão das comunicações ferroviárias e as doenças já era debatida. O médico mineiro Belisário Penna (1868-1939), por exemplo, fez duras críticas aos empreendimentos ferroviários realizados, principalmente, em sua terra natal. Na obra *Minas e Rio Grande do Sul: o estado da doença e o estado da saúde* , de 1918, o médico afirmou que as ferrovias eram fruto da “politicagem mineira” e que, apesar de em toda parte serem percebidas como “elemento de progresso e crescimento econômico”, em Minas, seriam meios para favorecer oligarquias, produzir malefícios como déficits constantes e impactos na saúde dos mineiros. O médico reclamava da falta de uma estrutura sanitária centralizada no estado. Afirmou que os governos municipais tinham maior autonomia diante de questões relacionadas à saúde pública, salvo em casos mais graves como epidemias. No entanto, nem todas as câmaras municipais tinham recursos para desenvolver políticas públicas eficientes, por isso, as considerava “células doentes” que, por conseguinte, também adoeciam todo “o organismo”. Penna afirmou que não havia recursos para assistir a enorme massa de gente que acompanhava a penetração das estradas e “elevava o número dos depositários de germens perigosos”. A moléstia de Chagas e o impaludismo foram as doenças citadas por ele (Penna, 1918, p.21-28).

As ferrovias, portanto, além de cargas e passageiros, transportavam seres invisíveis que, ao se instalar nas superfícies que compunham o material rodante dos trens, percorriam grandes distâncias para dar continuidade ao seu próprio ciclo biológico e colonização de novas áreas. Bresalier (2012) destacou que o agente da gripe fazia parte de um grupo de patógenos que não podiam ser vistos com microscópios de luz ou estudados pelos métodos de cultura que tiveram tanto sucesso com bactérias no período. Doença microbiana, em 1918 a gripe ainda não tinha seu agente causador efetivamente identificado, existindo intensa discussão entre médicos e cientistas nessa época. Em 1931, o americano Richard Shope (1901-1966) conseguiu isolar pela primeira vez o vírus *influenza* do trato respiratório de suínos; e, somente em 1933, os investigadores britânicos Wilson Smith, Christopher Andrews e Patrick Laidlaw identificaram esse vírus, denominado *Myxovirus influenzae* , como o agente causador da gripe no ser humano (Rebelo-de-Andrade, Felismino, 2018, p.8).^1^

A transmissão do vírus pode ocorrer por contato direto (pessoa-pessoa) pela via respiratória, por meio de gotículas ou aerossóis expelidos por indivíduo infectado durante o ato de espirrar, tossir ou falar. Há também possibilidade de contágio por transmissão indireta – uma pessoa pode adquirir *influenza* ao tocar, com as mãos, uma superfície ou um objeto contaminado com o vírus e, em seguida, tocar os olhos, boca ou nariz ( Rodrigues et al., 2007 ; Oliveira et al., 2013 ). Considerando essas características, em particular, a alta capacidade de dispersão do vírus, argumentamos que a movimentação ferroviária em Diamantina desempenhou papel importante na determinação dos padrões de propagação da epidemia de gripe de 1918 na região.

Essa doença, alcunhada de “gripe espanhola”,^2^ teve entre suas manifestações clínicas letais a extrema cianose, que corresponde a pele e mucosas de coloração azul escura por má oxigenação, traduzindo falência dos pulmões infectados em transferir oxigênio para a corrente sanguínea, acompanhada de tosse e pneumonia (Abreu Jr., 2018, p.42). O exato número de óbitos causados pela epidemia de gripe de 1918 segue desconhecido, porque muitos locais afetados não possuíam registros de estatísticas de mortalidade ( Kolata, 2002 , p.17), mas estima-se que o número total de vítimas esteja entre vinte e cem milhões de pessoas.

Posto isso, o artigo analisa o episódio epidêmico de 1918 em Diamantina. Em um primeiro momento observa a consolidação dos planos de integração regional por meio da construção de um ramal ferroviário e as condições materiais que esse ramal ofereceu para o avanço da epidemia de gripe no município e região em 1918. Discorre também sobre o contato e as respostas da sociedade diamantinense à epidemia.

## O ramal ferroviário de Diamantina e a modernização do norte de Minas

O debate sobre a construção de uma ferrovia em Diamantina começou nas últimas três décadas do século XIX (Via Lactea, maio 1914, p.2), contudo, apenas no início do século XX um projeto com esse objetivo se consolidou. A demanda pela construção de uma ferrovia foi uma das estratégias às quais recorreram as elites diamantinenses com o objetivo de consolidar investimentos que exigiam escoadouros para a produção vinícola e têxtil ( Fernandes, 2005 , p.80-84). Os políticos locais, por sua vez, seriam privilegiados, uma vez que teriam prestígio em suas empreitadas eleitorais, sendo reconhecidos como agentes facilitadores daquela que se pretendia ser a maior obra urbana da cidade. Durante a Primeira República, a cidade elegia seis deputados na esfera estadual e se constituía como nono distrito no plano das eleições federais ( Martins, 2014 , p.250-258).

Com o auxílio da expressiva imprensa local (Goodwin Jr., 2007), as elites diamantinenses operacionalizaram o histórico discurso de isolamento regional com o objetivo de angariar aliados para a perspectiva de que a ferrovia seria o meio mais rápido e eficaz de promover a integração do norte de Minas ao restante do país. Esse discurso foi permeado por representações associadas às características biofísicas da serra do Espinhaço, a que o município está circunscrito. A área possui altitudes consideráveis, estendendo-se por mais de 1.200 quilômetros de Minas Gerais à Bahia ( Knauer, 2007 ).

A serra do Espinhaço está na interseção de três grandes biomas brasileiros, a saber: Mata Atlântica, Cerrado e Caatinga. Além disso, de importantes bacias hidrográficas do país: rio São Francisco, rio Jequitinhonha e rio Doce. O sítio da cidade de Diamantina possui altitudes que variam entre 1.100 e 1.350 metros ( Varajão, 2015 , p.45). Sob as coordenadas 18,25° de latitude sul e 43,60° de longitude oeste, onde se localiza a sede do município, a elevação média do terreno é de 1.250 metros acima do nível do mar (p.30). Recorrentemente, as condições precárias das estradas regionais foram associadas às características dessa serra, em particular à sua topografia. Até o início do século XX, as estradas no norte de Minas se limitavam a trechos abertos no período colonial ( Souza, 1993 , p.155). O artigo intitulado “Basta de sonhar”, do jornal diamantinense *O Jequitinhonha* , afirmava que a ferrovia seria o elemento civilizador que, ao “quebrar as montanhas”, permitiria à cidade recuperar “a energia [do período minerador] que lhe era própria” (Basta..., 8 maio 1904, p.4).

As condições serranas informavam não só que a cidade era de difícil acesso, mas também que se tratava de um ambiente com clima ameno. Na obra *Chorografia do município de Diamantina* , publicada originalmente em 1899, o intelectual diamantinense José Augusto Neves (1875-1955) louva as benesses do clima local. O autor afirma que a sede municipal de Diamantina tinha aspecto “alegre, com clima ameno e sadio”. Especificamente sobre o clima, Neves (1986 , p.136) afirmou que era “muito saudável, sendo o ponto mais quente os vales dos rios, mais ameno o planalto carrasquento e mais temperado o planalto diamantino”. Ele também destacou que na região não grassavam “epidemias e moléstias endêmicas” (p.136).

É importante salientar também, em termos do discurso de isolamento, que aspectos históricos do processo de colonização regional contribuíram igualmente para a representação da região como um espaço isolado. O modelo de administração implantado no distrito diamantino a partir de 1771, que visava garantir o controle e a segurança da mineração de diamantes, ficou na memória local como momento de grande repressão e violência realizadas pelas autoridades sobre toda a população, indiscriminadamente ( Furtado, 2012 , p.75).

Nem sempre, contudo, a percepção do isolamento apresentou-se de modo negativo. No início da atividade mineradora, por exemplo, o governo português tinha um entendimento predominante de que o difícil acesso a algumas regiões de Minas Gerais dificultaria o contrabando de minérios extraídos. Outro aspecto refere-se ao imaginário social acerca da identidade mineira (mineiridade), que se formou no período da mineração. Em geral, a perspectiva era a de que o território tornava os mineiros mais propensos à tranquilidade e à moderação, portanto, conciliadores natos. Essas características, especialmente no contexto republicano, eram apontadas como positivas. Além disso, devido à fama aurífera e à ideia de um rápido enriquecimento, predominava também uma visão de Minas Gerais como uma terra de pensamento liberal e que, justamente por ser isolada, entre as montanhas, seria reduto de uma identidade genuinamente brasileira. Portanto, o tom de valoração acerca do isolamento oscila, dependendo do período abordado. Em Diamantina, sua percepção em tom negativo se realçou quando, na região, a demanda por ferrovias fez-se crescente. Essa conjuntura tornou o conflito – real ou potencial – com o meio biofísico daquele espaço cada vez mais percebido como dramático, e a inauguração de um ramal ferroviário foi apontada como “o meio de remediar tamanho inconveniente que representava o isolamento da região” (Via Lactea, maio 1914, p.1).

Era fevereiro de 1902 quando, por meio do decreto n.4.377, se consolidou um projeto em vias de alcançar a cidade. Fruto da fusão e modificação de concessões liberadas para a construção de duas estradas de ferro no território mineiro em 1890, o projeto deu forma à estrada de ferro que também prometia ligar aquela porção de Minas ao litoral brasileiro (Brasil, 1902).

O decreto estabelecia a construção de uma ferrovia a partir de Vitória, no litoral do Espírito Santo, em direção ao norte mineiro, tendo como ponta de trilho o município de Diamantina. Criada a Companhia Vitória a Minas em 5 de agosto de 1902, foram iniciados os estudos visando alcançar o território diamantinense. Os estudos estiveram a cargo do engenheiro Emílio Schnoor (1855-1923). Na presidência da nova companhia estava o engenheiro João Teixeira Soares (1848-1927) e, entre os diretores, Pedro Nolasco. Os trabalhos de construção foram iniciados em 1903 em Vitória (Relatório..., 1903). O mapa a seguir mostra detalhes da rota planejada.

O projeto ferroviário previa a criação de uma rede regional de comunicações. Minas, a partir da EFVM, teria acesso ao litoral do Espírito Santo e, posteriormente, ligaria o município de Peçanha ao de Filadélfia, atual Teófilo Otoni. Deste último, teria ligação com a Estrada de Ferro de Caravelas, no litoral baiano. Também foi planejada a ligação com a EFCB, que já estava em território mineiro, com vistas a chegar às margens do rio São Francisco (Relatório..., 1903).

A EFCB avançava por Minas desde 1869, sendo a primeira ferrovia em território mineiro, quando ainda tinha por nome Estrada de Ferro Dom Pedro II ( Pimenta, 1950 , p.2). Duas estações dessa ferrovia foram inauguradas próximo a Diamantina. A primeira, em junho de 1904, na sede do município de Curvelo, e, em 1905, a segunda no distrito curvelano de Curralinho. Ante essa situação, as elites de Diamantina se percebiam cada vez mais próximas dos trilhos, fosse pela EFVM ou pela EFCB.

O ambicioso projeto, no entanto, não deu certo. Em 1908, quando a ferrovia já estava no território mineiro, com a ponta dos trilhos no quilômetro 313, na estação da cidade mineira de Cachoeirinha, à direita do rio Doce, os concessionários da empresa consideraram alterar o traçado da via-férrea ( Pimenta, 1950 , p.7). Essa mudança foi orientada pelos estudos produzidos pelo Serviço Geológico e Mineralógico do Brasil. Criado em 1907, esse órgão tinha entre suas principais funções a realização de estudos científicos sobre a estrutura geológica e os recursos minerais do país ( Figueirôa, 1997 ).

Os primeiros estudos do Serviço Geológico e Mineralógico do Brasil indicavam o município de Itabira, na Zona da Mata mineira, como uma das áreas do quadrilátero ferrífero – estrutura geológica cuja forma assemelha-se a um quadrado com extensão de 7.000km^2^ (Roeser, Roeser, 2010, p.33) –, área em que seria possível explorar quantidades exorbitantes de minério de ferro. Dessa feita, diante da maior rentabilidade econômica, os concessionários da EFVM consideraram alterar a rota que tinha Diamantina como destino. Assim, ocorreu. Em 1909, foram aprovadas as modificações no traçado da EFVM.

**Figura 1:**
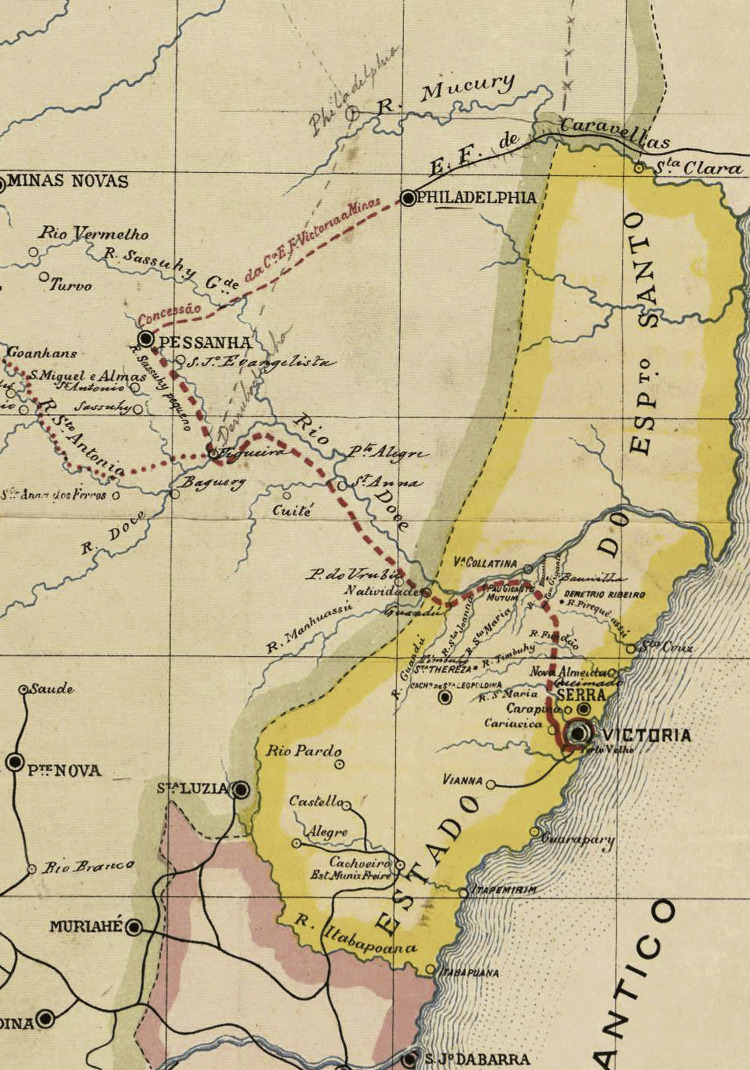
: Detalhe do traçado da Estrada de Ferro Vitória a Minas (Relatório..., 1903, Anexo)

A alternativa rápida e viável para que Diamantina fosse contemplada com trilhos seria a construção de um ramal que ligasse a cidade à EFCB, que já avançava na altura do município vizinho de Curvelo. Assim, em julho do mesmo ano, com mediação de políticos locais e de uma Comissão Popular Permanente fundada em Diamantina, foi autorizada a construção de um ramal ferroviário que sairia do distrito curvelano de Curralinho, atual município de Corinto, em direção a Diamantina.

A Comissão Popular Permanente, criada em 31 de janeiro de 1909, foi organizada em Diamantina num contexto em que os planos da EFVM de chegar à cidade pareciam declinar em favor de Itabira. Essa comissão lançou mão de vários recursos para chamar a atenção das autoridades para a questão ferroviária na cidade. O seu principal objetivo era envidar esforços para que pelo menos os trilhos da Central do Brasil, em Curralinho, chegassem a Diamantina. Para tanto, a comissão organizou encontros e manifestações nas ruas de Diamantina e de Belo Horizonte, trocas de ofícios com personalidades do norte mineiro, abaixo-assinados e a elaboração de relatórios com avaliações positivas sobre as características ambientais da região (Neves, s.d.). O diálogo com personalidades políticas locais, como Francisco Sá (1892-1936), também marcou a atuação da referida comissão.

Nascido na região, em Grão-Mogol, Sá foi nomeado, em julho de 1909, ministro de Viação e Obras Públicas pelo então presidente Nilo Peçanha (1867-1924). Uma vez ministro, Sá aproveitou a ocasião e se apressou em atender às demandas de seus conterrâneos. No mesmo mês em que tomou posse, foi apresentado ao presidente o decreto n.7.455, que alterava a concessão n.4.337, de fevereiro de 1902. O novo decreto tratava das resoluções sobre a linha Curralinho-Diamantina, definindo que “fica substituído o trecho de Sant’Anna dos Ferros a Serro, da Estrada de Ferro Vitória a Diamantina, pela de Curralinho, da Estrada Central do Brasil, à cidade de Diamantina”. O prolongamento de Curralinho a Diamantina seria realizado pela EFVM.

O jornal *O Norte* comemorou a assinatura do decreto. Na edição de 17 de julho, além de elogios a Francisco Sá, foram reproduzidos pelo menos 14 telegramas que teriam sido enviados à Comissão Popular Permanente e que elogiavam a postura desta em prol do prolongamento Estrada de Ferro Curralinho a Diamantina. Entre os remetentes estavam Pedro Mata, Carlos Otoni, Wenceslau Braz e Juscelino Barbosa. Garantida a construção do ramal da Central do Brasil até a cidade, a comissão passou a empreender novas batalhas, fosse observando, passo a passo, a construção que foi iniciada em 16 de outubro de 1909, fosse tendo choques diretos com o que poderia descarrilar a locomotiva antes mesmo de os trilhos serem colocados.

O traçado do futuro ramal de Diamantina foi apontado como sinuoso, entre espigões e grotas, contando com constante movimento de terras devido à inclinação lateral do terreno. O andamento das obras enfrentou dificuldades por causa das características do terreno atravessado, do clima e de alguns rios: das Velhas, Jabuticabas, Capim Branco, Pardo e Tabatinga (Relatório..., 1910, p.9). Além disso, foram constantes as reclamações relativas às condições precárias dos trabalhadores empregados na construção do ramal. A EFVM precisou enviar um fiscal para investigar denúncias das irregularidades. Os empreiteiros do ramal elevavam o preço dos gêneros alimentícios, e os salários dos operários eram baixos (A Idea..., 17 abr. 1910, p.2). Por conta desses aspectos, as obras só foram concluídas em 1914, com a inauguração da estação de Diamantina em 3 de maio.

O ramal ferroviário inaugurado em Diamantina em 1914 era bem diferente e mais modesto do que apontavam os primeiros estudos para sua construção. A nova ferrovia iniciou sua operação plena contando com oito estações e o total de 147km^2^ de extensão. As estações que compunham a linha, até a irrupção do episódio epidêmico de 1918, eram: Curralinho, Roça do Brejo, Santo Hipólito, Rodeador, Riacho das Varas, Baraúnas, Guinda, Diamantina (Relatório..., 1917, p.12-15).

Logo no início de suas atividades, foram recorrentes as reclamações sobre a prestação de serviços na linha, percebida como extremamente cara e lenta. Mesmo em 1909, no início de sua construção, já era manifestado o desejo de que a linha fosse encampada pela União e integrada à Central do Brasil. A demanda pela encampação foi reforçada pela crise econômica gerada pela Primeira Guerra Mundial (1914-1918) e partiu tanto de interesses diamantinenses quanto da própria Vitória-Minas.

Construído e administrado pela EFVM, mas conectado aos trilhos da Central do Brasil, o ramal de Diamantina foi descrito pelo jornal *Pão de Santo Antonio* (16 mar. 1918, p.1) como “a maior iniquidade administrativa do Brasil”. Toda essa situação contribuiu para a retomada do discurso de isolamento regional. Eram duas estradas diferentes, portanto, duas formas de administração também distintas. Esse aspecto acentuava a morosidade na prestação de serviços da linha. Mas as dificuldades não se limitavam aos aspectos burocráticos. O ramal, como a orientação de grande parte do traçado da EFVM, tinha a bitola métrica (1m).^3^ Esse aspecto técnico era considerado mais econômico e viável para uma zona que, desde seu início, foi percebida como sendo de baixa produtividade. Já a EFCB possuía um sistema de bitola mista ( Campos, 2012 ), essas diferenças estruturais causavam incômodos constantes como baldeações para troca de produtos e de carros de passageiros que duravam até 24 horas e tornavam as tarifas e passagens mais caras (Pão de..., 16 mar. 1918, p.2). A encampação, portanto, parecia imprescindível.

A operação da linha foi sempre deficitária, com vida econômica e tráfego precários. O crescimento econômico esperado não ocorreu, e a sensação de isolamento, reclamada pelas elites daquele espaço, não parecia mitigada. A encampação pela Central do Brasil ocorreu apenas em 1922 (Pão de..., 26 nov. 1922, p.3). A nosso ver, porém, a constatação da epidemia de gripe na cidade em 1918, antes mesmo de o ramal se tornar parte da EFCB, representou o cumprimento do propósito da ferrovia de integrar aquele sertão, visto que mesmo os incômodos vividos em grandes centros, como o referido episódio epidêmico, puderam atingir aquele espaço antes percebido como um dos mais abandonados de Minas Gerais.

Quando a epidemia forçou a ferrovia Central do Brasil a reduzir as suas atividades, o ramal de Diamantina permaneceu em funcionamento. O período da Primeira Guerra Mundial trouxe muitas dificuldades ao processo de amortização dos contratos internacionais da EFVM. Continuar as atividades seria, portanto, uma maneira de evitar maiores prejuízos às linhas Vitória-Itabira e Curralinho-Diamantina (Relatório..., 1919, p.25).

Era 1919 quando Ceciliano Abel de Almeida, chefe do tráfego da EFVM, agradeceu aos funcionários da companhia pela subordinação “nos dias difíceis, do surto da gripe”. Em suas palavras, no ano anterior, “só não foi alterado o serviço dos trens e das estações porque muitos, dedicadamente vinham cuidar das suas obrigações, quando já sentiam os primeiros sintomas da moléstia ou quando apenas entravam em convalescença” (Relatório..., 1919). Essa postura contribuiu para que mais indivíduos fossem expostos ao risco de contágio pelo vírus da doença.

## A viagem da gripe nos vagões da Central do Brasil

Verdadeiramente assombrosa foi a epidemia de gripe que irrompeu no Estado em fins de 1918. O Governo tudo envidou para socorrer a população do Estado, vítima dessa grande calamidade. ... Para a obtenção de médicos [bem] como de medicamentos o governo lutou a princípio com grandes dificuldades, devido à irrupção simultânea da epidemia em toda a parte ( Minas Gerais, 1919 , p.67).

Minas Gerais possuía a maior malha ferroviária do país (Batista, Barbosa, Godoy, 2012), e esse aspecto certamente influenciou a percepção declarada por Arthur da Silva Bernardes (1875-1955), presidente do estado, sobre a epidemia como fenômeno “simultâneo” em toda parte. A extensão ferroviária mineira em tráfego em 1918 era de 6.557,298km ( Minas Gerais, 1919 , p.105).

A linha Curralinho-Diamantina não estava isenta do contato com lugares onde a epidemia já estava ocorrendo. Embora fosse curta (147,5km), possuía ligação direta com a Central do Brasil, na época a segunda maior ferrovia do país (1.281,143km) ( Minas Gerais, 1919 , p.105).

A Central do Brasil partia da capital federal, Rio de Janeiro, e tinha como objetivo alcançar o rio São Francisco, na altura de Pirapora (MG). No mapa a seguir, de 1919, destacamos detalhes de sua linha-tronco por Minas Gerais. O itinerário era composto pelas cidades de Belo Horizonte (vermelho), Sete Lagoas (amarelo), Curvelo (azul) e seu distrito Curralinho (rosa) – deste último, partia o prolongamento até Diamantina (verde).

Na cidade do Rio de Janeiro, com 910.710 habitantes, cerca de 15 mil indivíduos morreram vítimas da epidemia e, pelo menos, 600 mil foram a leito, o equivalente a cerca de “66% da população local” ( Goulart, 2005 , p.105). Outra cidade no trajeto dos trens em direção a Diamantina era Belo Horizonte. As estimativas indicam que 27% da população belo-horizontina tenha sido atingida pela doença, o que representa cerca de “15 mil pessoas em um total de 55.709 habitantes” ( Silveira, 2008 , p.164).

Desse modo, partindo do Rio de Janeiro, passando por Belo Horizonte, em direção ao norte de Minas, o contato entre a gripe espanhola e a cidade de Diamantina seria inevitável. O material rodante do ramal diamantinense em 1918 era composto por quatro locomotivas, sete carros de passageiros, sete carros de bagagens, sete carros de correios e, ainda, 49 vagões (Relatório..., 1917, p.12-15). Os municípios de Curvelo e Diamantina estavam separados por oito estações. Com fluxo contínuo, os trens saíam do distrito curvelano de Curralinho às segundas, quartas e sextas-feiras, e saíam de Diamantina às terças e quintas-feiras e aos sábados.

**Figura 2:**
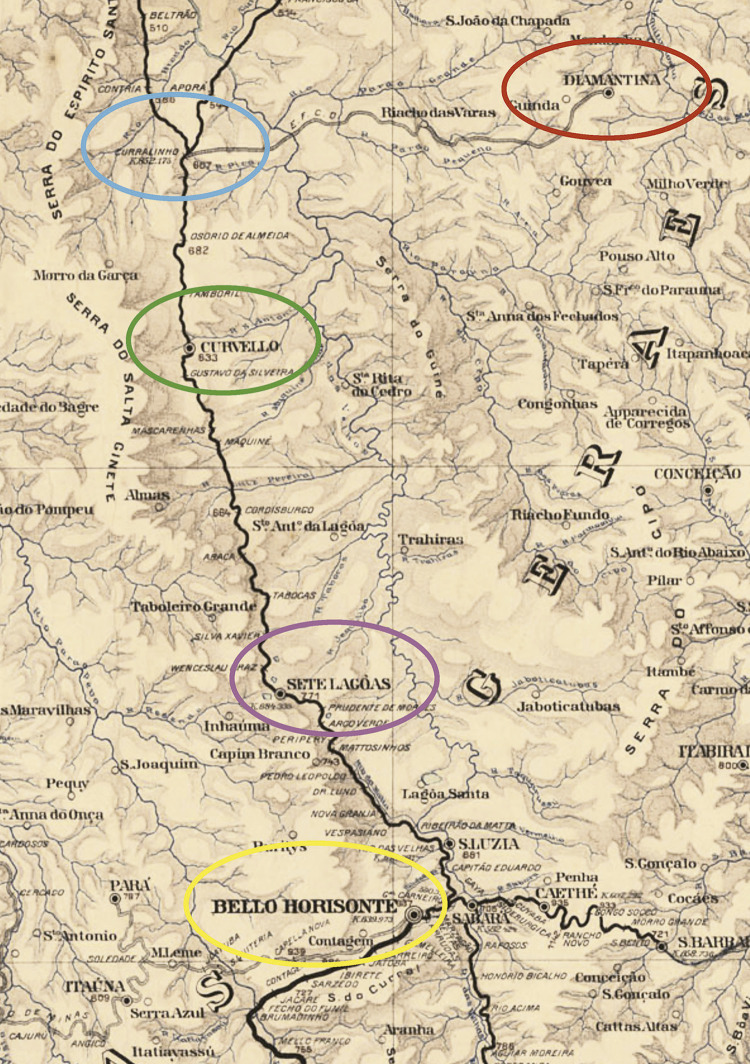
: Mapa com detalhe do itinerário da Central do Brasil e sua ligação com o ramal de Diamantina (Fonte: Carta..., 1919)

A linha Curralinho-Diamantina, em sua maioria, era composta por estações de quarta classe. Essas estações tinham dois quartos, armazém, agência, cozinha e pátio. Apenas na ponta de trilho, a sede municipal, foi construída uma estação de primeira classe. Essa possuía um armazém, uma biblioteca e telegrafia, dois quartos, sala de visitas, sala de jantar, cozinha, banheiro, plataforma e um salão de embarque ( Brasil, 1910 ).

A estação ferroviária da sede municipal de Diamantina, situada no largo Dom João, sua maior praça pública no período, foi um importante espaço de sociabilidades. Muitos eventos aconteciam ao seu redor, e em sua proximidade estava localizada outra importante instituição, o Seminário Episcopal. Alguns exemplos dos eventos que ocorriam na estação eram a chegada de figuras ilustres, missas, despedidas e procissões. A movimentação constante de pessoas chegou a mobilizar alertas recorrentes no jornal *Pão de Santo Antonio* (30 maio 1920, p.3), a saber: “Na estação do nosso ramal, pode se dar, a qualquer hora, um desastre evitável. A meninada ali pinta o sete, por um milagre, não houve ainda uma desgraça a lamentar-se”.

Em torno das estações houve incentivo ao comércio e à criação de uma estrutura para atender às demandas de passageiros (restaurantes, bares e hotéis). Em Diamantina, em 1918, funcionavam pelo menos três hotéis: Hotel do Comércio, Hotel Itambé e Hotel Diamantina e Gomes ( Senna, 1918 , p.613-647). A nova dinâmica implicava maior agilidade nos serviços urbanos, como correios e telégrafos, que também foram beneficiados com o advento da nova linha férrea.

A ferrovia alterou a distribuição das estradas de rodagem ao estimular a construção de rodovias que contribuiriam com a chegada de maior quantidade de produtos às estações para, assim, atender melhor a cidade e a região ( Martins, 2014 , p.152-170). Essas rotas e esses caminhos facilitariam também a chegada de mais pessoas às estações da linha.

Em 1918, Diamantina foi destacada, pelo anuário mineiro, como um dos maiores municípios do estado. No total, eram 17 distritos e uma população de 75 mil habitantes. A sede municipal, onde estava estabelecida a estação final do ramal, possuía uma população de 12 mil habitantes e abrigava importantes instituições como um arcebispado, a sede do terceiro Batalhão da Brigada Policial de Minas, as zonas de fiscalização de impostos federais e estaduais, a 11ª circunscrição eleitoral do estado e a nona circunscrição de obras públicas do estado e da Delegacia dos Terrenos Diamantinos. Contava também com dois colégios de instrução secundária (sendo um deles o seminário episcopal) e uma Escola Normal ( Senna, 1918 ).

Eram essas as condições de Diamantina quando a epidemia irrompeu. A estimativa é de que em 1918 a linha tenha transportado cerca de 16.130 pessoas (Relatório..., 1919, p.5). A ferrovia pode ter contribuído para a disseminação da doença na região por meio de passageiros infectados que viajaram na linha, sem necessariamente perceber a ameaça que representavam. Ou, ainda, o material rodante (vagões, locomotivas, carros de passageiros) e permanente (estações, caixas d’agua) daquele ramal poderiam abrigar em suas superfícies patógenos, como vírus, ampliando a sua disseminação. A estrada de ferro, ao expor a cidade a maior contato com o vírus da doença, deixou de representar apenas benefícios e progresso para aquele local.

## Encontros e despedidas: a epidemia na cidade

Inspirado no livro *A peste* , de Albert Camus, o historiador estadunidense Rosenberg (1992) nos apresenta a “dramaturgia das epidemias”, narrativa observável em experiências epidêmicas passadas que geralmente se caracteriza por ciclos de negação, ressignificação, resignação e esquecimento. O primeiro momento implicaria o reconhecimento gradativo da epidemia, uma vez que o reconhecimento do estado epidêmico ameaçaria interesses econômicos e específicos em geral. O segundo momento consistiria na elaboração de explicações para o fenômeno envolvendo elementos de diversas ordens (morais, religiosos e científicos) e na busca pelos “culpados”. O terceiro, por sua vez, implicaria negociações e ações por respostas, um processo que envolve solidariedade e atuação coletiva da sociedade. Por fim, o quarto implica o progressivo abrandamento do surto e a retrospecção sobre o ocorrido com o desejo de extrair algo a partir da trágica experiência que representou. Silveira (2008) apresenta um interessante balanço de estudos dedicados à história das epidemias a partir de duas abordagens: biológica, voltada para características como o agente patológico da doença, o meio ecológico, entre outros aspectos dessa ordem; e social, que, por sua vez, em perspectiva semelhante à de Rosenberg, abordaria os modos como as diferentes sociedades reagiram e interpretaram o fenômeno epidêmico. A autora destaca que estudos voltados para a visão social da doença revelaram que uma comparação entre as respostas e as reações das diversas sociedades que sucumbiram às epidemias demonstra uma estrutura narrativa observável, tal como a “dramaturgia” das epidemias proposta por Rosenberg. Contudo, essa dramaturgia – amplamente citada por historiadores, como aponta Souza (2007 , p.41) – define a estrutura do evento, mas não consegue abarcar a diversidade do contexto ou a complexidade da sociedade em que a doença se manifesta, por exemplo.

Posto isso, analisamos a manifestação do episódio epidêmico em Diamantina, onde os discursos sobre isolamento e salubridade local, dada sua localização estar circunscrita à serra do Espinhaço, acabaram sendo elaborados a partir das representações da referida serra. Entre o repertório desses discursos esteve a ideia de que, por conta das características do ambiente serrano, Diamantina sofreria menos com a epidemia de gripe, e isso não aconteceu. A “senhorita espanhola” visitou a cidade e, quando partiu, não deixou saudades.

A partir de fins de outubro de 1918, jornais diamantinenses começaram a publicar as primeiras notícias sobre a doença. O jornal católico *A Estrela Polar* , aos 27 de outubro, noticiou que a situação epidêmica seria consequência grave dos pecados humanos – portanto, um castigo divino. Em outra edição do mesmo ano, de 8 de novembro, o artigo “A lição dos acontecimentos” denominava a doença “maligna *influenza* ” e “tufão da morte”.

Em outros surtos epidêmicos ocorridos na história brasileira, a relação entre as enfermidades e os pecados humanos também esteve presente. Franco (2014) analisou a epidemia de cólera no Espírito Santo no século XIX, demonstrando como a “teoria divina” foi resultado da forma assustadora como a cólera avançava na região. Em busca do perdão divino, uma série de penitências, orações e procissões foi rigorosamente seguida pela sociedade.

Em Diamantina, pedidos de provisão para a realização de procissões para São Sebastião foram recorrentes como uma resposta à epidemia de 1918. O santo mártir é reconhecido como protetor dos enfermos. Costumeiramente, as festas em sua homenagem acontecem no mês de janeiro, mas verificamos a realização de procissões no município dedicadas ao referido santo em novembro de 1918. O padre Leopoldo Seabra (1918) , do distrito diamantinense Mercês do Arassuahy, atual cidade de Senador Modestino Gonçalves, solicitava à arquidiocese de Diamantina: “A vista da epidemia que está grassando, [os fiéis] pedem-me para promover uma procissão de São Sebastião, de penitência, pelo que peço a necessária bênção para promovê-la, enviando inclusa a garantia de 20 mil réis”. Segundo o jornal *A Estrela Polar* , aos 15 de dezembro, em Riacho das Varas, atual distrito diamantinense de Conselheiro Mata, foi realizada uma festa de penitência para o “glorioso mártir”. “Com grande acompanhamento de fiéis”, o evento contou com uma missa matinal e procissão que se dirigiu à estação da EFVM. Esses acontecimentos públicos, durante a epidemia que já circulava pela cidade, certamente sujeitaram mais pessoas ao risco de contaminação pela doença.

A chave explicativa que esteve relacionada às percepções religiosas representa uma das respostas da sociedade de Diamantina, essencialmente católica, às experiências que a epidemia desencadeou. Aspecto que confirma a afirmativa de Le Goff (1985) de que as doenças têm história e estão ligadas a estruturas sociais, instituições, representações e mentalidades de uma determinada época.

**Figura 3:**
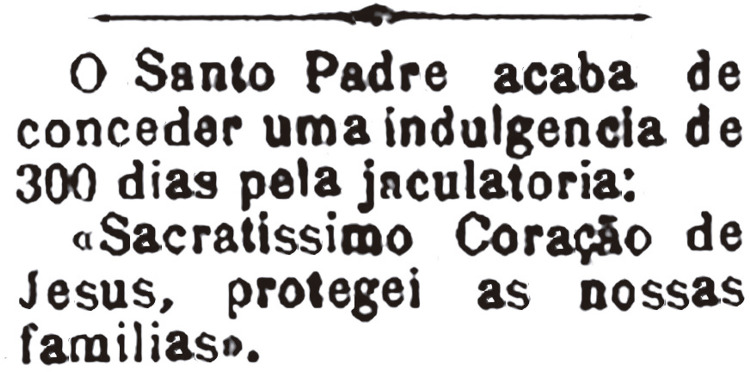
: Recorte da imprensa diamantinense divulgando oração de proteção contra a epidemia de 1918 (Fonte: Pão de..., 1 dez. 1918, p.1)

Além da abordagem religiosa, nos jornais locais há registros de outras respostas à epidemia. No jornal *A Estrela Polar,* o artigo intitulado “O transmissor da espanhola”, que inicialmente teria sido publicado na revista portuguesa *Brotéria* , discutia a semelhança entre a gripe espanhola e uma enfermidade identificada como “doença dos três dias”. A perspectiva era a de que o flebótomo, inseto da família dos *Psychodidae* , seria o vetor das duas doenças. Também foi recorrente nos jornais, além da vulgarização das controvérsias científicas acerca da epidemia que irrompia, recomendações sobre as melhores formas de tratar os sintomas da doença.

É importante ressaltar que, no período, foi intenso o debate sobre a origem e a natureza dos possíveis agentes infeciosos para a epidemia reinante. As discussões mais acesas ocorreram, principalmente, em torno do bacilo de Pfeiffer ( *Hemophilus influenza* ), que, desde o fim do século XIX, muitos acreditavam ser o agente causador da doença. Apenas na década de 1930, como já foi mencionado, o vírus *influenza* foi indicado como o agente viral causador da gripe no ser humano (Rebelo-de-Andrade, Felismino, 2018). Entretanto, desde a virada para o século XX muitos estudos indicaram a possibilidade de um vírus filtrável ser a causa da gripe humana, entre eles o do instituto de pesquisa britânico Medical Research Council ( Bresalier, 2012 ); essas pesquisas ganharam ênfase em 1918 e foram realizadas também no Brasil ( Bertucci, 2014 ).

O jornal *Pão de Santo Antonio* (1 dez. 1918, p.2) recomendava que a boca, a garganta e as fossas nasais fossem “constantemente e perfeitamente” lavadas, pois seriam “as principais, senão únicas entradas do micróbio ou micróbios, ainda não conhecidos, portadores da *influenza* ”. Para a lavagem da boca e garganta, seria necessário bochechar e gargarejar com folhas de eucalipto, canela, sal e água fervida. Para a limpeza das narinas, por sua vez, recomendava-se o uso moderado de “uma pitadinha” de rapé. Aspectos dessa natureza também são evidenciados na análise de outras ocorrências da epidemia no Brasil. Abrão (1998) , tratando do episódio epidêmico em Porto Alegre, salienta que certo oportunismo mobilizou alguns setores durante o período de epidemia, como os ganhos financeiros obtidos pelas indústrias de medicamentos, chocolates e cigarros.

Ainda nos jornais, médicos que prestavam serviços itinerantes divulgaram as vantagens de seus serviços. Segue uma narrativa que elucida bem esse cenário em Diamantina:

O dr. José R. Monteiro da Silva, ilustrado médico que há muitos anos se dedica ao tratamento das moléstias por meio de ervas medicinais, aconselha, contra a *influenza* , o seguinte remédio, que acho muito bom: folhas de eucalipto, de camará ou cambará, manga, pitanga, laranja-da-terra limão, alfavaca, louro, melão-de-são-caetano, flores de mamoeiro, flores de bananeira. Deite-se um punhado de cada uma dessas folhas e flores em um grande bule, e ponha-se de infusão por uma hora em 3 garrafas de água bem fervendo, tomando-se depois uma xícara de 2 em 2 horas. Os casos leves e benignos curam-se com essa infusão de ervas agasalhando na cama. Está muito aconselhado o tratamento pela essência de canela. Vou fazer uma denúncia inocente, anunciando que há um pé de canela no quintal do Dr. Telles de Menezes, um outro no de D. Josefina Felício e com certeza também na chácara do falecido Juca Neves, de saudosíssima memória (Pão de..., 3 nov. 1918, p.2).

O chá de ervas foi indicado como uma alternativa mais “fácil, barata e eficaz” às drogas da farmácia que, segundo o relato, estariam “caríssimas”. A assertiva seria recorrer às folhas que poderiam ser encontradas, inclusive, na casa de alguns médicos da cidade. Em Diamantina, no período, três farmácias, as instituições Motta & Prado, Pharmacia Horta e Gruta de Lourdes, puderam oferecer seus produtos que, vinculados a noções de cientificidade, chamavam para si a eficácia no combate à doença a fim de às mais clientes.

A primeira manifestação oficial sobre a epidemia em Diamantina ocorreu pela imprensa em 10 de novembro de 1918. Cosme Alves do Couto, agente executivo em exercício, cargo hoje equivalente ao de prefeito, frisou que seu discurso pretendia “desfazer o pavor causado pela pandemia de gripe”. Segue o texto:

A existência desta epidemia foi verificada aqui no dia 23 pelo médico do 3º batalhão onde se alastrou com rapidez, porém, de forma tão benigna que até hoje não se verificou um só óbito em nosso meio causado exclusivamente pela pavorosa *influenza* . Parece, Sr., Redator, que o clima de Diamantina é privilegiado contra o terrível mal, que assola em todos os cantos do Brasil, que deveria ser aconselhado pelos médicos para refúgio das pessoas timoratas e até dos convalescentes atacados em outros pontos do país (A Estrela..., 10 nov. 1918, p.3).

A lentidão e a negação do estado de doença não é um fenômeno neutro ( Goulart, 2005 , p.104-105). Alves do Couto procurou amenizar as manifestações da epidemia na cidade destacando a doença como de caráter benigno e o clima local como salubre, visto que o reconhecimento do estado epidêmico poderia contrastar com a imagem regional historicamente ostentada acerca da salubridade de seu clima. Essa questão parecia tão cara que, no final de seu discurso, Alves do Couto afirmou que, por conta de seu clima, Diamantina deveria ser aconselhada pelos médicos como refúgio de pessoas doentes vindas de outras partes do país. Para fundamentar suas afirmações acerca do caráter benigno da epidemia, Alves do Couto apresentou dados extraídos do registro de óbitos do cemitério da cidade, entre os dias 23 de outubro e 7 de novembro, a saber:

Outubro

29) Anna Pereira Gomes, 76 anos de idade, ‘sem assistência médica’.

30) Maria Theodora, 50 anos de idade, ‘sem assistência médica’.

31) Hermano de Siqueira, 24 anos de idade, pneumonia.

Novembro

03) João Inocêncio de Faria, nefrite

04) Maria Luiza, recém-nascida

04) Joaquim Salvador, preso na cidade, ‘gripe complicada’ com tuberculose

04) Benedito Ribeiro de Souza, ‘gripe complicada’.

06) Maria, recém-nascida (A Estrela..., 10 nov. 1918, p.3; destaques nossos).

A qualidade do ar do território de Minas Gerais foi recorrente nas representações sobre o estado. No final do século XIX muitas pessoas foram atraídas ao território mineiro em busca de tratamento para doenças como, por exemplo, a tuberculose (Marques, Silveira, Figueiredo, 2011, p.73). O entendimento de que locais montanhosos seriam mais saudáveis devido aos ventos frequentes derivava de teorias miasmáticas que, sinteticamente, compreendiam enfermidade e salubridade a partir da fixação ou deslocamento dos ares. Apesar da prevalência do paradigma da bacteriologia no início do século XX, concepções miasmáticas continuaram presentes nas ações de saúde e saneamento e na cultura popular, como revelado no discurso de Alves do Couto.

Apesar da ênfase do discurso oficial de que a salubridade local iria abrandar a enfermidade, há registros de ocorrências de doenças que desorganizavam o cotidiano daquela região antes mesmo do episódio epidêmico de 1918. Um exemplo pode ser observado em 1908, quando a Delegacia de Higiene de Diamantina adotou algumas medidas para conter o avanço da varíola, que teria sido detectada nos distritos de Rio Manso e Rio Preto, que atualmente correspondem às cidades de Couto de Magalhães de Minas e São Gonçalo do Rio Preto, respectivamente. As medidas de controle adotadas foram a requisição de praças para instalação de um cordão sanitário e o uso de “medicamentos e desinfetantes” (A Idea..., 29 nov. 1908, p.1). Esse exemplo, como a epidemia de 1918, indica que a retórica ostentada sobre a salubridade regional não podia ser sustentada – considerando, especialmente, o crescimento demográfico e os novos motes que se estabeleciam: abastecimento de água, rede de esgotos, insalubridade urbana e maior circulação facilitada pela linha ferroviária da EFVM.

A demora em reconhecer o avanço e o estado epidêmico em Diamantina, portanto, demonstra que, até aquele momento, nenhuma estratégia visando combater a epidemia havia sido preparada. Embora tenha sido destacada inicialmente com caráter benigno, a gripe avançou. Em 9 de novembro de 1918, isto é, um dia antes da manifestação do Poder Executivo municipal, uma carta foi enviada pela irmã Eugênia a dom Joaquim Silvério, arcebispo de Diamantina. Na correspondência foi apresentada a situação dos alunos do quarto ano, entre os quais “muitos” teriam sido “atacados pela epidemia”. Assim, segundo a religiosa, “seria mais conveniente que a distribuição dos diplomas” se realizasse em março de 1919. Posto isso, a irmã encerrou sua correspondência pedindo uma bênção e uma prece “para que nosso senhor nos proteja” (Arquidiocese..., 1918). A doença começava a desorganizar o cotidiano da cidade.

A subnotificação da doença pode ter sido um dos efeitos diretos da postura do Poder Executivo municipal. Além de a gripe ser uma doença comum, o discurso oficial pode ter contribuído para o fato de que, a princípio, pouca importância tenha sido dada aos primeiros casos ocorridos em Diamantina. Como consequência, as manifestações anteriores da doença podem nem mesmo ter sido registradas. Como destacou Silveira (2008) , a desorganização dos sistemas de registro oficial, bem como as dificuldades de diagnóstico e de atribuição segura da causa da morte, é observada na maioria dos estudos sobre a pandemia de 1918.

Reflexos dessa desorganização podem ser evidenciados nos dados constantes no livro de óbito disponível no Arquivo Eclesiástico da Arquidiocese e nos registros da Santa Casa da cidade, que não convergem. No livro de óbito de 1918, em outubro foram registradas 17 mortes em Diamantina. Dessas, pelo menos duas podem ser associadas à gripe de 1918, pois o diagnóstico apresentado como *causa mortis* era gripe e moléstia desconhecida. Em novembro, período mais intenso de convalescença da doença na cidade, do total de 73 mortes, 47 podem ser associadas à epidemia, uma vez que as causas apontadas foram: gripe intestinal, brônquio-pneumonia-gripal, *influenza* , gripe cardíaca, moléstia desconhecida e gripe com complicações. A partir de dezembro, os óbitos diminuíram (20 mortes), entre as quais pelo menos oito podem ser associadas ao episódio epidêmico.

Os registros da Santa Casa, por sua vez, apresentam dados divergentes dos já mencionados. O livro de entradas e saídas da Santa Casa de Diamantina, entre novembro de 1918 e fevereiro de 1919, registrou o total de 77 pacientes, dos quais apenas 47 foram diagnosticados com gripe. No livro, os indivíduos registrados eram todos do sexo masculino, e dos gripados ocorreram apenas três mortes.

No que diz respeito à gripe, no entanto, os números nunca são exatos. O caráter familiar da doença contribuiu para uma dissonância nos números apontados pelas fontes, por exemplo, para uma mesma cidade ou um mesmo hospital. Em Diamantina, uma justificativa primeira deve-se ao fato de que o livro de óbito registra apenas indivíduos que pertenciam à sede do município, ao passo que os registros da Santa Casa registram indivíduos oriundos de distritos municipais mais distantes e até mesmo outros municípios, como Curvelo e Montes Claros.

Além disso, declarações do médico Antônio Mota, diretor da Santa Casa, em edição de 8 de dezembro do jornal *Pão de Santo Antonio* , nos dão pistas sobre a ação daquela instituição diante do surto epidêmico na cidade e nos ajudam a compreender melhor os motivos na divergência dos números e como ocorreu o combate à doença no município. Segundo o médico, a Santa Casa teria estabelecido acordo com a Câmara Municipal para que a cidade não passasse pelas desgraças que outros centros vivenciavam. O diretor do hospital declarou que a instituição estava preparada para servir a trezentos gripados e devidamente aparelhada para enfrentar, com vantagem, a situação.

A pauta principal da entrevista com o diretor da Santa Casa tratou das reclamações de parte de população quanto à falta de atendimento e das recorrentes relações de óbitos verificados como “sem assistência médica”. Para Mota, a diminuta porcentagem de óbitos verificados no estabelecimento, totalizando seis mulheres e quatro homens, evidenciava ser infundadas as reclamações. Afirmou também que o pequeno coeficiente de mortalidade seria ainda menor caso se considerasse que as pessoas só procuravam o abrigo hospitalar quando já estavam bastante debilitadas.

Na ocasião, o diretor da Santa Casa informou ainda que, até o dia 2 de dezembro de 1918, a instituição havia recebido 232 gripados, entre os quais 120 eram militares. Segundo o diretor, a instituição gastou uma quantia superior a um conto de réis, e, diante de “despesas extraordinárias”, foi importante o auxílio do arcebispo dom Joaquim Silveiro de Souza, que prestou colaboração com “generosa doação de 200$00”. Afirmou ainda que o acordo estabelecido em novembro de 1918 com a Câmara Municipal possibilitou que as enfermarias da Santa Casa pudessem atender os enfermos, incluindo “alguns levados contra sua própria vontade, para serem tratados como pensionistas da câmara”. Como a situação pedia mais agilidade a fim de “evitar delongas nas entradas, foram suspensas todas as formalidades regimentais: a administração interna do estabelecimento, exercida por irmãs de São Vicente de Paulo, estava sempre pronta para receber os doentes” (Pão de..., 8 dez. 1918, p.3). Esse aspecto pode ter contribuído para a discrepância entre os dados já mencionados.

No registro de entradas e saídas da Santa Casa foram apontadas as respectivas profissões dos doentes acometidos pela gripe: lavradores, mineiros, carroceiros, jardineiros, pedreiros, padeiros, negociantes, carpinteiros e um foguista, sendo lavradores os mais recorrentes. O perfil indica, sobretudo, que se tratava de indivíduos pobres. O foguista, por exemplo, trabalhador responsável por operar as caldeiras das locomotivas a vapor, sofria constantes atrasos em seus salários. Há registro em Diamantina de que os trabalhadores da EFVM viviam em precárias condições, recebendo os seus salários com atraso de até quatro meses (Pão de..., 29 ago. 1915, p.2). Nesse sentido, corroboramos os estudos que indicam que a epidemia de gripe de 1918 afetou em maior medida pessoas mais expostas à pobreza ( Brito, 1997 ; Abreu Jr., 2018).

A vigência do acordo entre a Santa Casa e a Câmara Municipal estendeu-se até 5 de dezembro, quando terminou a intervenção municipal. O médico Eder Jansen de Mello, em entrevista ao *Pão de Santo Antonio,* em 1 de dezembro de 1918, referiu-se ao convênio como a alternativa mais prática para a cidade. Entretanto, lamentou que não tivesse ocorrido a criação de postos “fixos de socorros logo ao surgir do mal”. Segundo o médico, essas medidas “disseminando e regularizando os socorros médicos”, paralelamente à distribuição “de gêneros de primeira necessidade”, dariam maior eficiência no combate à epidemia. O médico agradeceu ainda o esforço da “abnegada classe farmacêutica”, que teria prestado todo o auxílio possível quando o “corpo médico” da cidade esteve “totalmente absorvido e posto rudemente à prova pela clínica domiciliaria”. Em suas palavras, foram os farmacêuticos que “dia e noite” trabalharam, “alguns até doentes, outros corriam domicílios, em socorro de enfermos sem assistência médica. Grande parte da população foi, pois, tratada por esses profissionais”.

Além das ações da Santa Casa, da Câmara Municipal e das farmácias, há registros de medidas de enfrentamento à epidemia por parte da superintendência do ramal ferroviário. Os relatórios da EFVM, especificamente para a linha Vitória-Itabira, indicam a atuação de dois distritos sanitários e dos serviços de socorro frente ao avanço da epidemia. A sessão dedicada à linha de Curralinho-Diamantina, porém, foi sempre sucinta e objetiva, além de não apresentar a mesma riqueza de informações como o espaço referente à linha-tronco. Essa negligência elucida claramente o propósito dos diamantinenses de que o ramal de Diamantina fosse logo encampado pela Central do Brasil.

Apesar das escassas informações, há registros de que a administração do ramal de Diamantina contou com os serviços dos médicos Lacerda Guimarães e, em Curralinho, Luiz Azambuja de Lacerda. Os médicos prestaram serviços a todos os necessitados, mesmo os estranhos à estrada, distribuindo medicamentos gratuitamente. Apesar desses esforços, certamente aquela ferrovia e os caminhos vicinais do município de Diamantina foram fundamentais para que a gripe de 1918 se proliferasse no município e na região, pois as atividades não foram suspensas.

O jornal *Pão de Santo Antonio* (1 dez. 1918, p.1) destacou que:

Quando foi do início da epidemia, cujas cortes aguerridas ocupam ainda hoje nosso território, ceifando, dia a dia, vidas por todos os títulos preciosas, o ilustre superintendente do Ramal de Diamantina, solicitou, telegraficamente, do governo do Estado, auxílio e socorro contra a peste que invadia a vasta região da Estrada. Que resposta lhe deram os grandes senhores do poder?

– Não ser possível atendê-lo.

A “enérgica e inteligente” atuação do superintendente ferroviário foi assertiva, na perspectiva do redator do jornal, pois “se dependessem da resposta do governo mineiro”, o pessoal da estrada não teria assistência médica, que foi fornecida pela diretoria da EFVM. O discurso indica que a resposta dos órgãos estaduais às necessidades municipais em tempos epidêmicos foi insatisfatória. No artigo foi destacado ainda que mesmo a Câmara Municipal, quando demandou atenção do governo mineiro, “sentindo-se sem defesa e meios de combate”, teve como resposta: “1/2 quilo de quinino e dois pequenos frascos com essência de Canela! Isto, para cerca de 20 mil pessoas” (Pão de..., 1 dez. 1918, p.1).

Além da ferrovia, caminhos e trilhas contribuíram para o avanço da doença, uma vez que eram rotas-padrão de circulação de pessoas. As caravanas que avançavam pela região sobre o lombo de burros e mulas, intituladas tropas de muares, por exemplo, também podem ter contribuído para a disseminação da doença. Segundo Martins (2014) , mesmo após o advento ferroviário, até meados do século XX, essas caravanas transportaram a maioria dos gêneros de abastecimentos consumidos em Diamantina. Somadas ao movimento das tropas, destacamos ainda as procissões e festas religiosas que não pararam de ocorrer no município mesmo durante a epidemia de 1918. Posto isso, é compreensível a manifestação da doença em outros distritos do município de Diamantina, servidos ou não pela ferrovia.

Em Mercês de Araçuaí, que não contava com estação da EFVM, a gripe grassava desde outubro e teria “atacado todos os lares”. Segundo o jornal *Pão de Santo Antonio* (15 dez. 1918), a doença se manifestou com caráter benigno, e, para sorte dos habitantes da localidade de poucos recursos, “apenas 4 ou 5 pessoas faleceram devido complicações”. Em Riacho das Varas, com estação da linha Curralinho-Diamantina, a gripe também foi destacada como benigna, tendo causado apenas um caso fatal, “mesmo assim por complicação com outras moléstias”. No distrito de Curralinho, o médico Zozimo Ramos Couto registrou 59 gripados.^4^ Sebastião Pereira da Luz foi “acometido de assalto pela sorrateira ‘gripe’ no distrito diamantinense de São Gonçalo do Rio Preto” (Pão de..., 8 dez. 1918, p.3, 15 dez. 1918, p.3; A Estrela..., 8 dez. 1918, p.3).

De Curvelo, sede do entroncamento da Central do Brasil, o padre Thiago em correspondência enviada a dom Joaquim Silvério, em 23 de novembro de 1918, desejava ao arcebispo saúde diante da doença que “grassa[va] por toda a parte”. E continuou: “Aqui em Curvelo não somos dos mais infelizes, os casos são muitos, mas em geral de caráter benigno”. Em outro município, no Serro, que fazia ligação com Diamantina pela estrada do Gavião, outra correspondência para o arcebispo, datada de 6 de dezembro daquele ano, informava que os serranos estariam “a braços com a gripe”. Havia cerca de cinquenta vitimados e um morto. E a medida de urgência da Caridade no Serro foi a organização de um hospital, na casa dos Ottoni, cedida pela Câmara Municipal. A prioridade era o “tratamento dos que nem casa tinham”.

Era início de dezembro de 1918 quando a epidemia começou a entrar em franco declínio na cidade. Aos poucos o cotidiano era retomado. O Tiro de Guerra, por exemplo, que havia suspendido suas atividades a partir do dia 10 de dezembro, retomou seu funcionamento regular. Em 15 de dezembro no *Pão de Santo Antonio* foi publicado que a “senhorita espanhola muito chorosa partia sem deixar saudades”. O desejo era o de que uma “impetuosa rajada de vento a destruísse, ou a levasse aos infinitos”. Contudo, ocorrências da gripe também foram registradas em 1919, sobretudo no mês de janeiro.

Em 1º de dezembro de 1918 o jornal *Pão de Santo Antonio* publicou uma pequena nota afirmando que os campos começavam “a ser frequentados pelas apreciadoras das gabirobas”, e seria esse um sinal evidente de que já dizia adeus a “maligna pandemia”. A esperança nas condições serranas era mais uma vez reforçada, e a epidemia de 1918 estava sendo relegada ao esquecimento.

## Considerações finais

No artigo, relacionamos a propagação da gripe de 1918 às condições específicas do ramal ferroviário de Diamantina, que, transportando mercadorias e pessoas, buscou cumprir o propósito de integrar aquela cidade a outras regiões do país. As características biofísicas da serra do Espinhaço impulsionaram a elaboração da imagem de um sertão isolado para aquela porção do norte de Minas Gerais. Observamos, contudo, que durante o episódio epidêmico, as condições serranas foram valoradas positivamente sendo indicadas como os elementos que tornariam brando o episódio epidêmico e garantiriam, também, o “envio de doentes de outras localidades para a região”. Entretanto, apesar do discurso oficial de cidade salubre, a epidemia atacou o cotidiano diamantinense, que, diante da incapacidade das autoridades estaduais de oferecer suporte em relação ao avanço da doença, teve a prestação de socorro deixada a cargo de atores locais.

O avanço da epidemia de gripe contribuiu para afirmar que a ferrovia teria cumprido o seu propósito de integrar aquele sertão. Esse episódio, quatro anos após a inauguração do ramal, expôs a natureza perigosa da tão reclamada integração regional. A modernização chegava àquele sertão pelos trilhos percebidos como solução para o diagnóstico de atraso e isolamento. Contudo, os trens trouxeram mudanças ambientais e problemas de saúde pública como a chegada, em fins de 1918, da epidemia de gripe. Ao informar sobre os riscos que a ferrovia recém-inaugurada em Diamantina representou para a epidemia de gripe de 1918, nosso estudo mostra como são imbricadas as relações entre modernização, mudanças ambientais e proliferação de doenças. Dessa forma, contribui com a reflexão sobre como futuras epidemias podem se espalhar.

Outra contribuição refere-se ao esforço de contar a história do norte de Minas em termos diferentes, por meio da história da saúde e das doenças, uma vez que a memória do auge da mineração ainda é o forte motor da história regional. Nosso estudo, além de contribuir com a historiografia regional, procura conectá-la à historiografia nacional ao considerar que a construção das ferrovias brasileiras no início do século XX esteve acompanhada da busca por uma identidade nacional cuja característica principal seria a modernidade. No entanto, como a experiência de Diamantina evidencia, esse processo não teve apenas os efeitos desejados. Nos trilhos do almejado progresso também embarcaram a devastação da natureza e as doenças epidêmicas.

## NOTAS

1 O vírus, pertencente à família *Orthomyxoviridae* , possui RNA de hélice única e é classificado nos tipos A, B ou C, de acordo com perfis antigênicos característicos. O vírus do tipo A pode infectar humanos e alguns animais (aves, suínos, cavalos, focas e baleias) e está implicado em episódios epidêmicos e pandêmicos; o vírus do tipo B infecta exclusivamente humanos, está ligado a surtos moderados; e o tipo C é endêmico, acomete humanos e suínos sem potencial epidêmico, associa-se com manifestações da doença de forma leve ou assintomática. Entre as principais características do vírus *influenza* está a sua alta transmissibilidade, principalmente do tipo A ( Spickler, 2005 ; Andrade et al., 2009 ; Da Costa, Merchan-Hamann, 2016).

2 Mesmo considerando os diferentes nomes atribuídos à doença − bronquite purulenta, pneumônica, febre dos três dias, morte púrpura, gripe brasileira etc. ( Alvarez et al., 2009 ) −, inclusive nas discussões antes de a maioria da comunidade científica internacional de 1918 reconhecer que a doença epidêmica era gripe, a denominação gripe espanhola ou *influenza* espanhola foi difundida mundialmente ( Echeverri Dávila, 1993 ).

3 Bitola é a distância entre as faces internas dos trilhos.

4 Referimo-nos ao distrito diamantinense de Curralinho, e não ao distrito curvelano homônimo de onde partia o entroncamento da EFVM com a Central do Brasil.
